# Durability of SrTiO_3_–TiO_2_ eutectic composite as a photoanode for photoelectrochemical water splitting

**DOI:** 10.1039/d3ra07927a

**Published:** 2023-12-05

**Authors:** Katarzyna Kolodziejak, Jaroslaw Sar, Konrad Wysmulek, Krzysztof Orlinski, Piotr Piotrowski, Michal Gajewski, Dorota Anna Pawlak

**Affiliations:** a Lukasiewicz Research Network – Institute of Microelectronics and Photonics Al. Lotnikow 32/46 02-668 Warsaw Poland; b Centre of Excellence ENSEMBLE3 Sp. z o. o. Wolczynska Str. 133 01-919 Warsaw Poland katarzyna.kolodziejak@ensemble3.eu; c Faculty of Chemistry, University of Warsaw Pasteura 1 02-093 Warsaw Poland

## Abstract

The idea of employing sunlight – a virtually inexhaustible source of energy – to catalyze various chemical reactions or generate electrical current is intensively studied nowadays. Here, we describe a method for testing photoelectrochemical (PEC) stability developed using the example of photoanodes from an SrTiO_3_–TiO_2_ eutectic composite. Eutectic composite stability measurements were carried out in long-term cycles: 0.5, 1, 2, 5, 10, 20 and 50 h of constant electrode operation (total of 88.5 h). After each cycle, cyclic voltammetry, electrochemical impedance spectroscopy, reflectance, roughness, SEM/EDS microstructure analysis and the content of Sr and Ti ions in the applied electrolyte solution were examined. The initial value of the photocurrent density was 1.95 mA cm^−2^ at a potential of 1.5 V *vs.* Ag/AgCl in a pH 2 electrolyte environment and under 6 suns of illumination it increased almost four times, reaching 7.22 mA cm^−2^ after a total of 88.5 h of PEC stability cycles. Due to the better catalytic properties of TiO_2_, this phase degrades faster, causing an increase in the roughness of the electrode surface. At the same time, reflectance of the photoanode active layer dropped from around 35% to 15%. The investigated method of PEC material testing can be applied in areas beyond photoelectrochemical water splitting, such as chemistry, photovoltaics, sensing and others.

## Abstract 2

Here, we describe a method for testing photoelectrochemical stability developed using photoanodes from an SrTiO_3_–TiO_2_ eutectic composite. The initial value of the photocurrent density was 1.95 mA cm^−2^ at 1.5 V *vs.* Ag/AgCl and increased to 7.22 mA cm^−2^ after 88.5 h of stability cycles.

## Graphical abstract

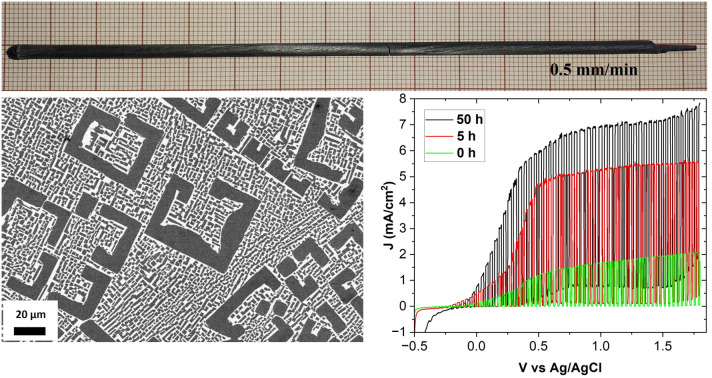

## 1. Introduction

Many techniques of obtaining hydrogen gas from water using sunlight exist today. One of the most promising is photoelectrochemical (PEC) splitting due to many advantages over other methods. These advantages include: (i) ability to carry out the process in ambient conditions (at room temperature and pressure);^1^ (ii) the hydrogen and oxygen evolution reactions take place at different electrodes, which eliminates mixing of these gases;^2^ (iii) preparation of photoelectrodes is usually very simple. Methods of PEC hydrogen production have been developed for over 50 years.^3^ At first, the light absorption layer of the electrodes comprised bulk materials, in particular single crystals.^4^ Promising conversion efficiencies of H_2_ and O_2_ generation were obtained through various doping, which extended the useable range of the solar spectrum. The breakthrough came with employing dye – sensitized mesoscopic materials in PEC cells, proposed by O'Regan and Gratzel.^5^ Nanostructures functionalized with organic dyes are the most commonly-investigated group of materials for solar-driven water splitting; but multijunction^6–8^ and organic^9,10^ electrodes are also showing promising results. Such nanomaterials exhibit relatively high conversion efficiencies due to their extended surface areas and good absorption properties. TiO_2_ heterojunctions can be a good example of this type of composite. Zhang *et al.* obtained high PEC performance by extending the absorbance range spectrum through the localized surface plasmon resonance (LSPR) effect in the prepared electrodes with Au NPs on the surface of flower-like TiO_2_.^11^ Efficient sensitization of TiO_2_ was achieved by sequential ionic layer absorption and reaction (SILAR), which allowed absorption to be extended from 3.21 eV (pure TiO_2_) to 1.98–2.16 eV (depending on the level of BiOI on TiO_2_).^12^ Again SILAR was used to obtain a heterojunction of TiO_2_ nanotube arrays/Bi_2_S_3_ quantum dots, which led to a minimization of the band gap to 1.3 eV, and thus to a 3.8 greater photocurrent density compared to bare TiO_2_.^13^ Almost 4 times higher photocurrent was obtained by creating a p–n junction of *p*-ZnO quantum dots and *n*-TiO_2_ by a solvothermal method. Thanks to the ZnO/TiO_2_ junction, the electrode showed greater electron–hole separation and thus increased the charge carrier lifetime.^14^ However, such materials suffer from high recombination rates of charge carriers and low stabilities. On the other hand, highly stable oxide compounds have wide band gaps, which enables absorption only in the UV range and a narrow visible range. A material which simultaneously exhibits high efficiency, durability, low cost and ease of manufacture is yet to emerge.^15^ Most currently developed materials exhibit only one of these properties.

Recently, we have shown the possibility of utilizing eutectic composites in PEC water splitting^16–19^ taking advantage of their ability to combine various material phases into one solid composite. Additionally, significant advantages of eutectic composites result from (i) high crystallinity (as single crystals),^20^ (ii) sharp interfaces between the component phases,^21^ (iii) the possibility of selecting different components enabling broadband absorption (UV-Vis), (iv) the use of phases that are difficult to achieve or otherwise unavailable, and (v) the ability to tune eutectic properties depending on requirements. The reaction, which leads to splitting of water molecules on semiconducting materials under solar irradiation, is associated with generation of charge carrier species: electrons and electron holes. The electron–hole pairs exhibit high redox potentials. This fact is exploited in photocatalytic processes such a PEC hydrogen production and photocatalytic CO_2_ reduction or degradation of organic compounds.^22^ However, in reality only a fraction of charge carriers react with the active material instead of the target medium (electrolyte solution), leading to degradation of material and loss of photocatalytic properties. Solar-driven catalysis is often carried out with an applied electrical bias, in an aggressive environment of an electrolyte solution which is either highly acidic or basic.

The stability of photoelectrodes in the PEC system is one of the crucial parameters because unstable materials affect the solar energy conversion efficiency. The stability of devices used for PEC water splitting is defined as the ability of the system to maintain the solar energy conversion efficiency at a steady level with predetermined tolerance over extended time periods. The fact that the water splitting reaction in PEC cells is carried out under solar light irradiation, together with an externally-applied bias, leads to a highly corrosive environment for the photoactive material. Thus, achieving high electrode stability, especially of the photoanode, is not easy and is being intensively studied. For example, Dias *et al.*^23^ investigated a hematite photoanode prepared by spray pyrolysis over 1000 h of sunlight exposure and showed a stable photocurrent density of 0.94 mA cm^−2^ at 1.45 V *vs.* RHE. On the other hand, TiO_2_ nanotube arrays with SrTiO_3_–TiO_2_ hetero-nanoparticles obtained by hydrothermal reaction showed no drop of hydrogen production generation rate and a stable photocurrent of 1.91 mA cm^−2^ at 0.3 V *vs.* SCE (saturated calomel electrode) after 25 h.^24^ Similar stability was reported by Bashiri *et al.*^25^ when they investigated a SrTiO_3_@TiO_2_@Fe_2_O_3_ nanorod heterostructure and recorded stable hydrogen production during its 25 h operation time. Similarly, a heterostructured Cr-doped SrTiO_3_–TiO_2_ photoanode retained a stable photocurrent density (4.05 mA cm^−2^*vs.* 0.6 V SCE) over 2 h of continuous working.^26^ Additionally, it was shown that a SrTiO3–TiO_2_ eutectic photoanode improved photocurrent response by 50% up to 8.5 mA cm^−2^ (at 1.5 V *vs.* NHE) after 30 h of continuous operation under light. The increased photocurrent was a result of higher absorbance due to increased surface area of the electrode. To summarize these various studies on the PEC electrode material stability: it is clear that there is a lack of a robust method that would provide a fair comparison of the properties of different materials, preparation methods and determination of long-term stability in photocatalysis processes.

In this work, we propose a universal method for PEC electrode stability investigation which can enable easier comparison of various materials. The method is based on following changes in the photoanode active material, such as: (i) the level of generated photocurrent; (ii) reflectance; (iii) concentration of photocorroded elements in the electrolyte; (iv) surface roughness after a defined number of operating cycles of PEC operation. We investigated the durability of a SrTiO_3_–TiO_2_ eutectic-based photoelectrode during 88.5 h of operation as a photoanode in PEC water splitting. The prepared photoanode revealed an improvement of photocurrent density, reaching its highest value of 7.22 mA cm^−2^ at 1.5 V *vs.* Ag/AgCl after a PEC stability test. The effects of the ongoing stability test were examined by scanning electron microscopy, reflectance measurements, profilometry, inductively coupled plasma mass spectrometry and PEC analysis.

## 2. Experimental

### 2.1. Crystal growth and material characterization

**Fig. 1:**
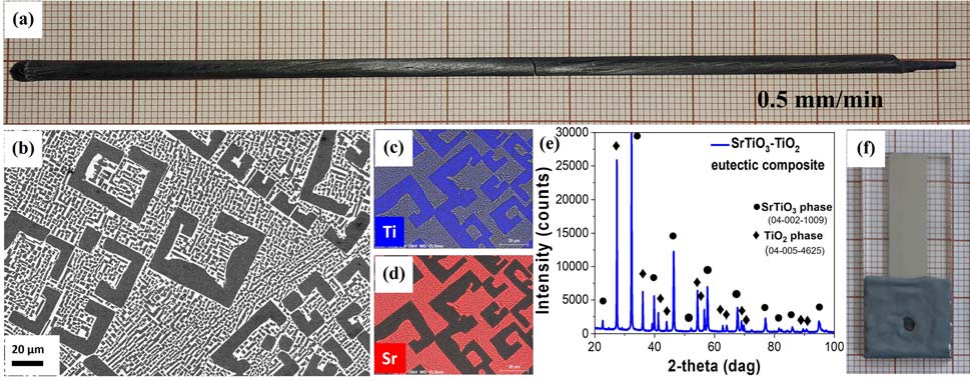
Directionally solidified SrTiO_3_–TiO_2_ eutectic composite as an active material in PEC water splitting. (a) As-grown rod obtained by the μ-PD method with 0.5 mm min^−1^ pulling rate; (b), SEM image presenting the composite microstructure; (c) and (d) EDS maps showing the distribution of Ti and Sr in the composite; (e) powder X-ray diffraction demonstrating two phases in the eutectic: TiO_2_ in the form of rutile and tausonite SrTiO_3_ phase; (f) image of the electrode with the SrTiO_3_–TiO_2_ eutectic composite as the photoelectrochemically active material.

The SrTiO_3_–TiO_2_ eutectic composite rod was grown (Fig. 1a) from 23 mol% SrO and 77 mol% TiO_2_ composition.^27^ The raw materials were prepared by mixing high-purity SrCO_3_ (Alfa Aesar; 99.99%) and TiO_2_ (Alfa Aesar; 99.99%) oxide powders. It was obtained by the micro-pulling-down method, μ-PD, in a nitrogen atmosphere with 0.5 mm min^−1^ pulling rate. The grown rod was 120 mm long with a 3 mm diameter. The μ-PD has been demonstrated to be a useful technique for quick material growth, and has been shown to enable fabrication of materials that find applications in various fields: optoelectronics;^28,29^ photonic crystals;^30–32^ metamaterials;^33–36^ THz materials;^37,38^ and SERS detection.^39,40^ X-ray powder diffraction (XRD) qualitative phase analysis of the SrTiO_3_–TiO_2_ eutectic composite was performed on the as-grown samples using a Rigaku SmartLab 3 kW diffractometer. The device was equipped with a high-speed 1D silicon strip (D/teX Ultra 250) detector and filtered Cu Kα (*λ* = 1.5418 Å) radiation. The diffraction patterns were measured in the Bragg–Brentano reflection geometry (*θ*/2*θ* scan) in the angle range of 10°≤ 2*θ* ≤ 100° with a scanning step of 0.01° and a speed of 2° per minute. Qualitative phase analysis was performed using the database PDF-4 + 2021 (ref. 41) and the PDXL2 Data Analysis Software supplied by Rigaku. Microstructure characterization was determined with a scanning electron microscope (SEM) Auriga Cross Beam Workstation (Carl Zeiss) equipped with an energy-dispersive spectroscopy detector (EDS) for chemical composition analysis (mapping). The reflectance measurements of the sample active surfaces before and after each long-time PEC analysis were performed using a CRAIC Technologies20/20 PV microspectrophotometer with an area of investigation of 100 × 100 μm and spectral range of 300–1000 nm. The photoanode roughness after PEC cycles was determined using a Veeco Dektak 150 surface profilometer. The total content of titanium and strontium ions in the electrolyte solution after PEC analysis was analyzed by an inductively-coupled plasma mass spectrometer (ICP-MS), NexION 300D ICP Mass Spectrometer, PerkinElmer SCIEX, Norwalk, CT, USA. The ICP-MS measurements employed a calibration curve with external standards, which were prepared using 1% nitric acid and titanium and strontium standards.

### 2.2. Photoanode preparation

SrTiO_3_–TiO_2_ rods were cut perpendicularly to the growth direction by a wire saw to form 1 mm thick slices. Slices were ground and polished to create *c.a.* 30 μm thick plates with a mirror-like surface. After that, the plates were glued to fluorine-doped tin oxide (FTO) coated glass substrates using silver epoxy paste (Elpox Sc 515 AMEPOX) which served, at the same time, as the ohmic contact layer. Silver paste was cured at 150 °C for 2 h in air at the hotplate. Afterwards, the excess of silver was covered by epoxy resin to avoid unwanted redox reactions during PEC analysis. The final electrode thickness, before the start of the stability test, was 31 μm with an active surface area of 0.04 cm^2^. The active surface area was measured using a Nikon Eclipse LV150 optical microscope.

### 2.3. PEC and stability measurements

The PEC measurements under illumination and dark conditions employed a CHI – 660D potentiostat (CHI Instruments) using: (i) open-circuit potential (OCP) to assess the stability of the system without irradiation and quality of the developed electrical contact; (ii) cyclic voltammetry (CV) to estimated intensity of photocurrent densities with an applied potential between −0.5–1.8 V and a 10 mV s^−1^ scan rate; (iii) amperometric current – time to test stability of electrodes in cycles (0.5, 1, 2, 5, 10, 20 and 50 h, total time of 88.5 h) under irradiation (600 mA cm^−2^ from a Solar Light 150 W xenon lamp). A three-electrode configuration placed in electrolyte solution in a Teflon/PTFE cell with a quartz window was used as a PEC analysis setup. A SrTiO_3_–TiO_2_ eutectic sample with thickness of 31 μm served as a working electrode; a silver/silver chloride (Ag/AgCl) electrode as the reference; and a platinum electrode as the counter electrode. Electrochemical impedance spectroscopy (EIS) was performed at 0 V in the frequency range of 1 MHz to 1 Hz using a 5 mV amplitude signal, both in dark and under illumination conditions. All PEC measurements were performed in electrolyte solution of 95% H_2_SO_4_, Na_2_SO_4_ and deionized water with pH 2 measured by a Seven Multi Mettler Toledo pH-meter. The irradiation intensity of the xenon lamp was calibrated using a Solar Light PMA 2144 Class II pyranometer.

## 3. Results and discussion

### 3.1. Crystal growth and eutectic composite characterization

In order to present a common method for judging the electrode durability and hydrogen production stability, we used the SrTiO_3_–TiO_2_ eutectic composite that has been previously demonstrated to form an efficient PEC photoanode.^18^ The material for investigation was prepared as described previously and is illustrated in Fig. 1, including the as-grown rod with 0.5 mm min^−1^ pulling rate and the microstructure of TiO_2_ precipitates embedded in the SrTiO_3_ matrix^42^ showed by SEM and energy dispersive spectroscopy (Fig. 1a–d). X-ray diffraction (Fig. 1e) confirmed the presence of a SrTiO_3_ phase in the form of tausonite (04-002-1009) and a TiO_2_ phase in the form of rutile (04-005-4625).^42,43^Fig. 1f shows a working electrode prepared from the demonstrated SrTiO_3_–TiO_2_ eutectic composite, which has been used for the stability test in this work.

In order to investigate the durability of the photoanode, we performed in parallel several measurements during the study to define a new methodology. The new methodology is based on changes in the photoanode active material, such as: (i) the level of generated photocurrent; (ii) reflectance; (iii) concentration of photocorroded elements in the electrolyte; (iv) surface roughness after a defined number of cycles of subsequent operation in PEC.

### 3.2. Roughness and microstructure analysis

**Fig. 2:**
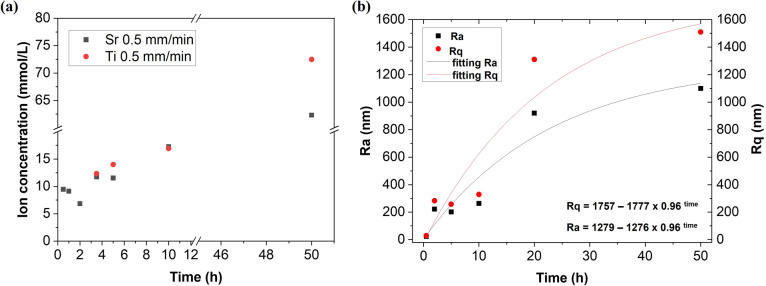
Photocorrosion of the SrTiO_3_–TiO_2_ electrode active material studied during 50 h of PEC water splitting operation: (a) amount of photocorroded Sr and Ti ions in the electrolyte used for PEC stability tests; (b) changes in the photoelectrode surface roughness characterised by measured Ra and Rq roughness parameters, together with fits.

In order to investigate the photocorrosion of the electrode active material, we studied the concentration of the photocorroded elements in the electrolyte using ICP-MS. The total content of Ti and Sr ions in the electrolyte measured by ICP-MS after each cycle was, on average, increasing with time. The first measurable Ti content was recorded after 5 h of cycles and then increased with subsequent cycles. The first four recorded Sr contents seemed to be at the limit of the method since the value was not affected by the working time or the eutectic surface was still robust enough. Finally, after 50 h of cycles, a higher concentration of Ti than Sr was observed in the electrolyte (Fig. 2a). Titanium ions can enter the electrolyte from both phases: titanium oxide and strontium titanate.

As the next step in the investigation of the photocorrosion of the electrode active material, we studied the surface roughness using a profilometer. The electrode surface quality can be expressed by two parameters: the arithmetic average roughness (Ra) and the root-mean-square roughness (Rq) as amplitude parameters of the deviation of the profile from the mean line. The parameters were estimated by scanning 1 mm_2_ of the SrTiO_3_–TiO_2_ electrode surface area. Results presented in Fig. 2b show an increase in the Ra and Rq parameters after subsequent stability cycles, corresponding to an increase in roughness. The experimental results were fitted using exponential functions.

**Fig. 3:**
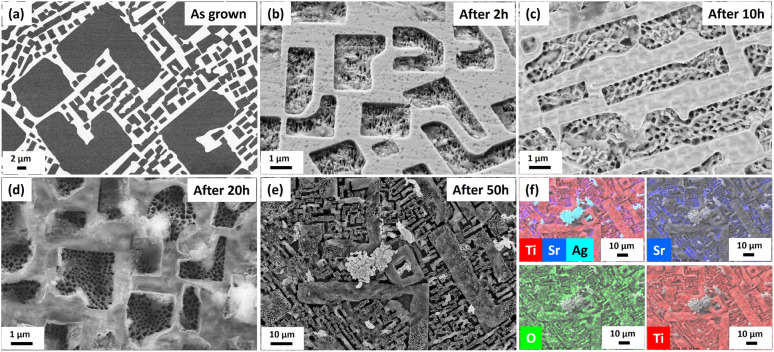
SrTiO_3_–TiO_2_ eutectic photoanode surface changes over time during PEC water splitting. (a)–(e) SEM images of the eutectic microstructure after continuous working time of the electrode – as grown, after 2, 10, 20 and 50 h respectively; (f) EDS maps showing the presence of Ti, Sr, O and Ag elements after 50 h working time on the electrode surface.

In parallel to the above, we investigated the surface roughness with scanning electron microscopy (SEM). It is strongly visible in the SEM images, mostly in the TiO_2_ phase which is suffering from the PEC process (Fig. 3b–e). The difference between the levels of individual phases deepens with progressive test time, starting from initial, optically polished active surface of the sample (Fig. 3a) to 2, 10, 20 and 50 h. After 50 h of PEC testing, the presence of the third phase can be observed on the sample surface (Fig. 3e). EDS map analysis revealed, in addition to the presence of Sr, Ti and O, Ag inclusions coming from the adhesive layer used in the tested SrTiO_3_–TiO_2_ photoanode (Fig. 3f). The presence of silver on the top of the photoanode proves that the process of degradation of the working electrode has begun.

### 3.3. Reflectance measurements of SrTiO_3_–TiO_2_ photoanode

**Fig. 4:**
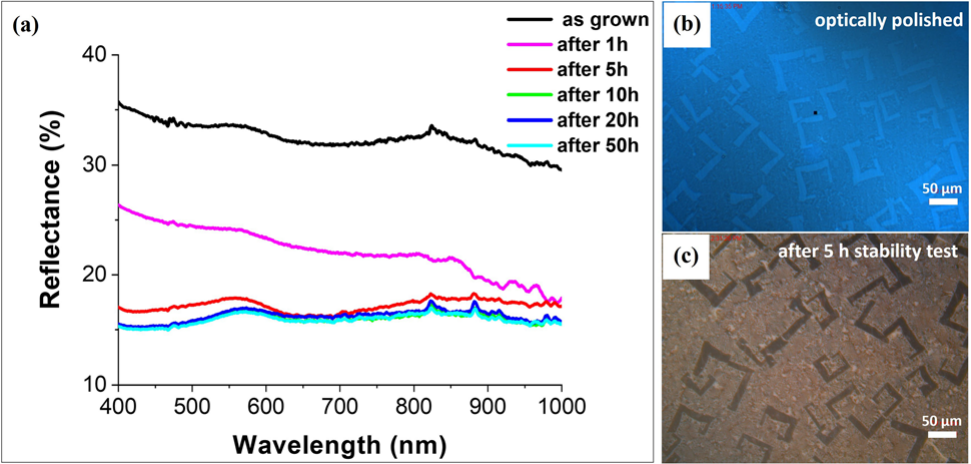
Changes of the SrTiO_3_–TiO_2_ electrode reflectance after successive cycles of PEC measurements. (a) Reflectance changes starting from the as-grown optically polished sample to the same sample after 50 h of operation; (b) and (c) microspectrophotometer images of the photoanode surface during reflectance measurements at the beginning and after 5 h of the PEC stability test.

As the next step, we used measurements of the electrode surface reflectance in order to study the surface roughness. Reflectance spectra were recorded in the 400–1000 nm wavelength range and showed a decrease in the value of the measured light reflection after the PEC stability measurement cycles (Fig. 4a). This is consistent with the observed increase in the roughness of the electrode in relation to the length of its continuous operation. Irregularities on the electrode surface cause light scattering and thus reduction of reflectance. The initial reflectance value of about 35% decreased down to 15% after 50 h of continuous operation. Fig. 4b and c show the initial mirror-like surface of SrTiO_3_–TiO_2_ and its surface after 5 h of stability test, respectively.

### 3.4. PEC and stability measurements

**Fig. 5:**
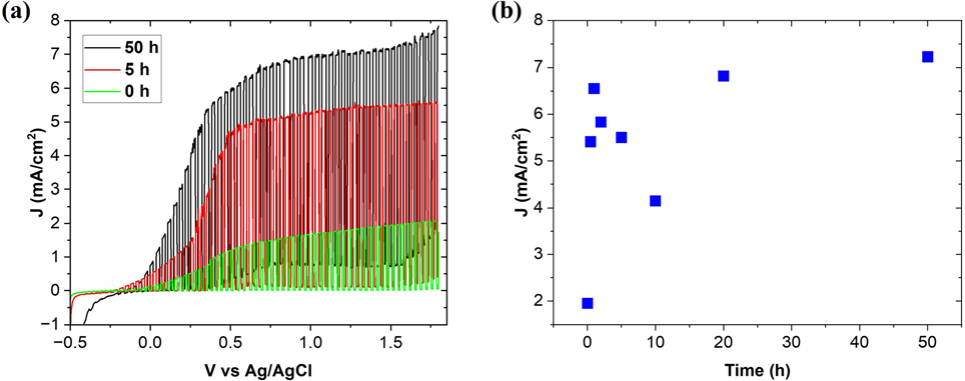
The evolution of generated photocurrent during continuous PEC operation. (a) The cyclic voltammetry (CV) results for the initial electrode, and after 5 and 50 h of operation under switching on and off the light, (b) changes of the photocurrent density with the operation time, measured for all stability test cycles at 1.5 V at Ag/AgCl.

PEC analysis and stability tests were carried out with cycles of: 0.5, 1, 2, 5, 10, 20 and 50 h of continuous operation at the applied voltage of 1.23 V (*vs.* Ag/AgCl) in the whole Xe-lamp spectrum range. Firstly, the open-circuit potential (OCP) was recorded to check the ohmic connection within the entire cell. The initially stable OCP value decreased under the light, confirming the photosensitivity and the n-type nature of the investigated electrode.^44,45^ After that, cyclic voltammetry (CV) analysis was carried out under modulated lighting (the light source was cyclically turned on and off) to investigate the possible presence of undesirable reactions (not related to photocatalysis). The current density values in the absence of lighting (dark currents) started to increase at the voltage of 1.65 V (*vs.* Ag/AgCl), which means that water electrolysis took place at this voltage and photoelectrolysis should occur before this value. Fig. 5a shows examples of CV scans performed before stability testing after 5 and 50 h of continuous operation. The initial photocurrent density increased from 1.95 to the maximum value of 7.22 mA cm^−2^ at 1.5 V *vs.* Ag/AgCl. This photocurrent density is still lower than the 8.5 mA cm^−2^ value reported previously for the SrTiO_3_–TiO_2_ eutectic after 30 h of operation^.^^17^ However, the starting thickness of that eutectic active layer was only 18 μm, which is much lower when compared to this work, 31 μm. The thicker active layer itself provides worse electrochemical response (longer charge carrier diffusion distance and higher resistivity).^46^ Additional improvement of the photocurrent is expected for further optimization of the electrode thickness or progressed PEC operation. It should also be noted that following the initial increases in photocurrents, after the next 2, 5 and 10 h cycles, the photocurrent values decreased to 4.15 mA cm^−2^ and then started to increase again. This can be explained by surface poisoning by residuals of etched TiO_2_ and SrTiO_3_, which block the surface for effective PEC process. Such a blocked surface was not photoelectrochemically active because of darkening. Moreover, detached particles were present in the solution but there was not chemical bonding with the electrode surface allowing transfer of the photogenerated electron–hole. Additionally, EDS analysis detected the Ag signal at the electrode surface. The reduced thickness of the eutectic electrode caused by etching led to the formation of the gap between the electrode surface and epoxy resin and exposure of the silver from the ohmic contact. Similarly to the residuals of SrTiO_3_ and TiO_2_, Ag particles blocked the electrode surface. Nevertheless, such a significant change in the overall value of photocurrents can be explained primarily by the increase of the working electrode active surface, leading to the creation of more active sites for light absorption and electrolyte adsorption. In addition, the overall thickness of the photoactive material decreased as a result of the continuous electrode operation. The summary of changes in photocurrent density after each PEC cycle is shown in Fig. 5b.

### 3.5. Electrochemical impedance spectroscopy (EIS)

**Fig. 6:**
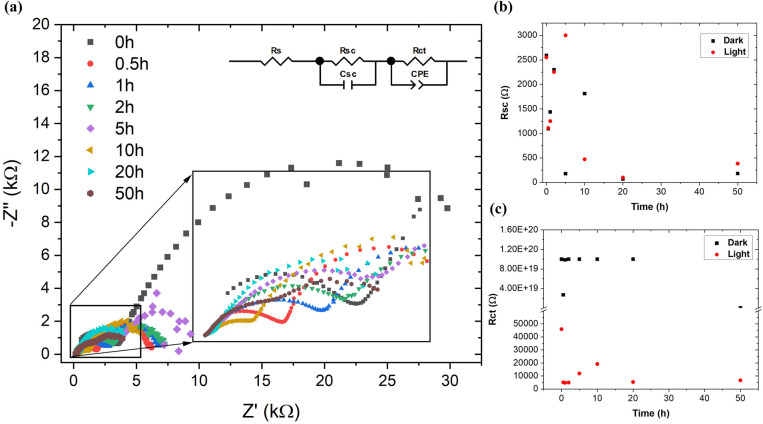
Electrochemical impedance spectroscopy: (a) Nyquist plots under illumination and equivalent circuit model (inset), (b) changes in semiconductor resistance (*R*_sc_), (c) changes in semiconductor–electrolyte charge transfer resistance (*R*_ct_) after successive times of the stability test.

In addition to PEC analysis, EIS measurements were performed when the light was turned on and off. The experimental results of EIS measured under illumination were fitted using the equivalent circuit model shown in Fig. 6a. The most important components of the model were the R–C associated with the semiconductor SrTiO_3_–TiO_2_ represented by the resistance (*R*_sc_) and capacitance of the capacitor (*C*_sc_) and R-CPE associated with the charge transfer between the semiconductor and the electrolyte (*R*_ct_ – charge transfer resistance, *Q*_dl_ – capacitance of the double layer).^47^ The fit results are summarized in Table 1. The total resistance to charge transfer decreases as the sample is illuminated. The main factor contributing to the decrease in total resistance was *R*_ct_.

**Table 1:** The results of the experimental data fitting to the proposed model of the equivalent circuit

Time (h)	Light conditions	*R*_s_ (Ω)	*R*_sc_ (Ω)	*C*_sc_ (F)	*R*_ct_ (Ω)	*Q*_dl_ (MF s^(*n* − 1)^)	*n*
0	Dark	800	2592	0.61 × 10^−8^	1.0 × 10^20^	1.14	0.85
	Light	576	2550	0.29 × 10^−8^	46 × 10^3^	4.42	0.68
0.5	Dark	657	1095	1.33 × 10^−8^	0.27 × 10^20^	2.55	0.93
	Light	547	1109	0.85 × 10^−8^	5.1 × 10^3^	6.96	0.76
1	Dark	723	1437	2.72 × 10^−8^	0.99 × 10^20^	3.65	0.92
	Light	766	1250	3.23 × 10^−8^	4.8 × 10^3^	6.43	0.83
2	Dark	657	2298	3.41 × 10^−8^	1.0 × 10^20^	5.03	0.89
	Light	645	2250	3.23 × 10^−8^	5.0 × 10^3^	8.61	0.78
5	Dark	353	179	20.7 × 10^−8^	1.0 × 10^20^	15.9	0.71
	Light	325	3000	22.9 × 10^−8^	12 × 10^3^	19.6	0.61
10	Dark	636	1813	19.6 × 10^−8^	1.0 × 10^20^	16.1	0.73
	Light	346	471	14.4 × 10^−8^	19 × 10^3^	53.2	0.53
20	Dark	203	70	80.7 × 10^−8^	1.0 × 10^20^	33.5	0.73
	Light	203	98	37.4 × 10^−8^	5.4E × 10^3^	29.1	0.70
50	Dark	117	180	27.8 × 10^−8^	4.2 × 10^17^	63.2	0.56
	Light	135	384	269 × 10^−8^	6.7 × 10^3^	67.0	0.44

The generated photo-holes migrate towards the semiconductor–electrolyte interface, while the photoelectrons move into the semiconductor. Photo-holes are involved in the oxidation of electrolyte ions. At the same time, no significant changes in the resistance values *R*_sc_ and *R*_ct_ were observed (Fig. 6a and b).

## 4. Conclusions

In this paper, we proposed a method for testing the stability of photoanodes for PEC water splitting. This method includes the study of: (i) the level of saturated photocurrents; (ii) the amount of light reflected from the surface of the active material; (iii) the concentration of Sr and Ti ions in the electrolyte; (iv) changes in surface roughness and (v) observation of the surface after stability cycles of progressive operation of the photoelectrode in intense illumination conditions. The PEC stability measurements based on a SrTiO_3_–TiO_2_ eutectic composite photoanode, were carried out over long time cycles starting from: 0.5, 1, 2, 5, 10, 20 and up to 50 h. The initial value of the photocurrent density of 1.95 mA cm^−2^ at the potential of 1.5 V *vs.* Ag/AgCl (in the electrolyte environment with pH = 2) increased almost four times to the value of 7.22 mA cm^−2^ after a total of 88.5 h of PEC stability cycles. The roughness of the active surface of the tested electrode, measured by profilometry, increased. It was additionally confirmed by a decrease in reflectance from about 35 to 15%. The higher concentration of Ti ions compared to Sr was measured using ICP-MS. The method of photoanode stability testing presented here can become a very practical tool for comparing the properties of electrodes made of different materials and determining their long-term usefulness in photocatalysis processes. The dissemination of this research method will contribute to accelerated stability testing, which is extremely necessary in the perspective of the development of this scientific field. Furthermore, this method can be used in the areas of material durability analysis with long operating cycles in the fields of science such as fuel cells, catalysis, nanotechnology or photovoltaics.

## Author contributions

K. K.: Conceptualization, methodology, investigation, project administration, writing – original draft preparation. J. S.: Methodology, investigation, writing – original draft preparation. K. O.: Methodology, investigation. K. W.: Investigation. P. P.: Methodology. M. G.: Methodology. D. A. P.: Conceptualization, writing – review and editing, resources, supervision.

## Conflicts of interest

There are no conflicts to declare.

## Supplementary Material
